# Draft Whole-Genome Sequence of the Fluorene-Degrading Sphingobium sp. Strain LB126, Isolated from a Polycyclic Aromatic Hydrocarbon-Contaminated Soil

**DOI:** 10.1128/genomeA.00249-18

**Published:** 2018-04-12

**Authors:** Floriana Augelletti, Julien Tremblay, Spiros N. Agathos, Ben Stenuit

**Affiliations:** a Earth & Life Institute, Laboratory of Bioengineering, Catholic University of Louvain, Louvain-la-Neuve, Belgium; b National Research Council Canada, Energy, Mining and Environment, Montreal, Quebec, Canada; c School of Biological Sciences and Engineering, Yachay Tech University, San Miguel de Urcuquí, Ecuador

## Abstract

We report here the draft whole-genome sequence of a fluorene-degrading bacterium, Sphingobium sp. strain LB126. The genes involved in the upper biodegradation pathway of fluorene are located on a plasmid, and the lower pathway that generates tricarboxylic acid cycle intermediates is initiated by the *meta*-cleavage of protocatechuic acid that is chromosomally encoded.

## GENOME ANNOUNCEMENT

Sphingobium sp. strain LB126 (formerly identified as Sphingomonas sp. [1, 2]) can use fluorene as a sole source of carbon and cometabolize other polycyclic aromatic hydrocarbons (PAHs) (3). Due to its biodegradative capabilities and metabolic funneling of different aromatics, strain LB126 is a promising candidate for bioaugmentation and valorization of lignin-derived monoaromatics as functionalized platform molecules in biorefineries.

Strain LB126 was kindly provided by Natalie Leys (Belgian Nuclear Research Center, Mol, Belgium) and grown in a mineral medium (4) containing 0.5 g liter^−1^ fluorene. DNA was extracted using the DNeasy PowerMax soil kit (Qiagen, Carlsbad, CA). The whole genome of strain LB126 was sequenced using the PacBio RS II sequencing platform (Pacific Biosciences, Menlo Park, CA). A 20-kb SMRTbell library was prepared and sequenced on one single-molecule real-time (SMRT) cell at the Génome Québec Innovation Centre (McGill University, Montréal, Canada). The *de novo* assembly was carried out using the Hierarchical Genome Assembly Process (HGAP)/Quiver protocol in SMRT Portal version 2.3.0.140936.p5 (5). The sequencing generated 734,729,152 bp from 98,542 subreads, with an average subread length of 8,425 bp. The final assembly accounts for 5,627,691 bp distributed in 14 contigs, with 130-fold coverage of the genome and an *N*_90_ value of 192,496 bp. Two contigs correspond to 2 circular chromosomes with lengths of 3,357,979 bp (Chrlb1, with 3,207 coding sequences [CDSs]) and 1,181,476 bp (Chrlb2, with 1,003 CDSs) and G+C contents of 64% and 63.2%, respectively. Three contigs correspond to circular plasmids, i.e., pSNA1 (109,558 bp), pSNA2 (88,783 bp), and pSNA3 (33,859 bp). Four contigs correspond to linear plasmids, i.e., pSNA4 (538,953 bp), pSNA5 (192,496 bp), pSNA6 (76,261 bp), and pSNA7 (41,257 bp). The assembled sequence data were annotated using the NCBI Prokaryotic Genome Annotation Pipeline (6). The whole genome contains 5,299 CDSs, 3 rRNA operons (2 in Chrlb2), and 57 tRNA genes. The 16S rRNA genes of strain LB126 shared 100% identity with those of the *rrn* operons of Sphingobium chungbukense DJ77 (7).

Strain LB126 degrades fluorene via the 9-hydroxyfluorene pathway to form *o*-phthalic acid (OPA) and protocatechuic acid (PCA) as intermediate products. pSNA1 harbors the genes involved in the upper fluorene biodegradation pathway, including the *fln* operon (*flnA1A2BDE* genes [4]), which generates OPA that is transformed to PCA by the *pht2345* genes (8). Chrlb2 contains the genes for the *meta*-cleavage pathway of PCA (2) that leads to pyruvate production (*fldABCDEFTUVWXYZ*) (9). The genome annotation reveals that Chrlb2 contains genes encoding enzymes involved in the metabolism of other aromatic compounds, such as phenol, benzoate, and catechol (e.g., *catRABC* gene cluster [10]). Moreover, plasmids pSNA4 and pSNA5 harbor multiple genes linked to heavy-metal resistance, such as the copper resistance operon *copABCD,* the cobalt-zinc-cadmium resistance protein CzcA, or an arsenic efflux pump protein. The presence of these metal resistance genes suggests that Sphingobium sp. strain LB126 could be a relevant microorganism for the bioremediation of sites cocontaminated with aromatics and heavy metals.

### Accession number(s).

This whole-genome shotgun project has been deposited in DDBJ/EMBL/GenBank under the accession number NETV00000000. The version described in this paper is the first version, NETV01000000.
